# Chemokine Receptors CCR6 and CXCR3 Are Necessary for CD4^+^ T Cell Mediated Ocular Surface Disease in Experimental Dry Eye Disease

**DOI:** 10.1371/journal.pone.0078508

**Published:** 2013-11-04

**Authors:** Terry G. Coursey, Niral B. Gandhi, Eugene A. Volpe, Stephen C. Pflugfelder, Cintia S. de Paiva

**Affiliations:** Ocular Surface Center, Dept. of Ophthalmology, Cullen Eye Institute, Baylor College of Medicine, Houston, Texas, United States of America; Wayne State University, United States of America

## Abstract

CD4^+^ T cells are essential to pathogenesis of ocular surface disease in dry eye. Two subtypes of CD4^+^ T cells, Th1 and Th17 cells, function concurrently in dry eye to mediate disease. This occurs in spite of the cross-regulation of IFN-γ and IL-17A, the prototypical cytokines Th1 and Th17 cells, respectively. Essential to an effective immune response are chemokines that direct and summon lymphocytes to specific tissues. T cell trafficking has been extensively studied in other models, but this is the first study to examine the role of chemokine receptors in ocular immune responses. Here, we demonstrate that the chemokine receptors, CCR6 and CXCR3, which are expressed on Th17 and Th1 cells, respectively, are required for the pathogenesis of dry eye disease, as CCR6KO and CXCR3KO mice do not develop disease under desiccating stress. CD4^+^ T cells from CCR6KO and CXCR3KO mice exposed to desiccating stress (DS) do not migrate to the ocular surface, but remain in the superficial cervical lymph nodes. In agreement with this, CD4^+^ T cells from CCR6 and CXCR3 deficient donors exposed to DS, when adoptively transferred to T cell deficient recipients manifest minimal signs of dry eye disease, including significantly less T cell infiltration, goblet cell loss, and expression of inflammatory cytokine and matrix metalloproteinase expression compared to wild-type donors. These findings highlight the important interaction of chemokine receptors on T cells and chemokine ligand expression on epithelial cells of the cornea and conjunctiva in dry eye pathogenesis and reveal potential new therapeutic targets for dry eye disease.

## Introduction

Tear dysfunction is one of the most prevalent eye conditions with reported prevalence ranging from 2–14.4% [1]–[7]. Patients with tear dysfunction typically experience intermittent to constant eye irritation, light sensitivity and blurred/fluctuating vision. Chronic dry eye can decrease quality of life in afflicted patients [8], and in some cases result in functional and occupational disability. Various treatment options are available; however, none of these target a specific biological pathway. Thus, understanding the pathogenesis of the disease may lead to new or improved therapeutic options that may vastly increase positive outcomes for patients.

It has been known for several years that dry eye disease (DED) is not simply a disease of decreased tear production but has a pathogenesis rooted in a T cell-mediated autoimmune response [9]. Although a complete understanding of the pathogenesis of this response has not been fully elucidated, there is increasing evidence that CD4^+^ T cells, specifically Th1 and Th17 cells, are major immune mediators of the disease [10], [11]. Our previous studies have shown that Th1 cells promote conjunctival squamous metaplasia and induction of apoptosis of conjunctival cells via the production of IFN-γ [10], [12]. IFN-γ also induces the loss of mucus-secreting goblet cells (GC) in the conjunctiva [10]. There is also evidence that Th17 cells are involved in pathogenesis via IL-17-induced (in conjunction with TNF-α and IL-1) production of matrix metalloproteinases (MMP) -3 and -9 that results in corneal epithelial barrier disruption [11].

The involvement of Th1 and Th17 cells in DED lead us to examine the migration of CD4^+^ T cells from the regional lymph nodes to the ocular surface (OS). Chemokines and their receptors serve as the central mediators coordinating localization of immune cells to specific tissues in order to execute an immune response. Th1 cells express the chemokine receptor CXCR3 (along with CCR5) that binds three IFN-γ-inducible chemokines: CXCL9 (MIG), CXCL10 (IP-10) and CXCL11 (I-TAC). The inducible nature of these chemokines by the prototypical Th1 cytokine, IFN-γ, suggests an amplification loop exists in which recruited Th1 cells, via production of IFN-γ, induce higher expression of CXCR3-binding chemokines that recruit additional Th1 cells to the site of inflammation. There is considerable evidence for the role of CXCR3 and CXCR3-binding ligands in many acute and chronic inflammatory and autoimmune diseases, such as asthma, rheumatoid arthritis, multiple sclerosis, and psoriasis [13], [14]. However, the role of chemokine receptors and their ligands is not fully understood in immune responses at the ocular surface. It is known that elevated concentrations of CXCL9, -10, -11 have been detected in the tears of dry eye patients [15]. Increased production of CXCR3 and CXCL-9, -10, and -11 have been observed in the ocular surface and increased frequency of CXCR3^+^ and CCR5^+^ T cells has been detected in draining lymph nodes of mice with experimental dry eye induced by subjecting them to desiccating stress (DS) [16], [17]. These findings suggest that lymphocyte homing to the ocular surface in dry eye is regulated by a chemokine/chemokine receptor network.

CCR6, expressed by Th17 cells and T regulatory cells (Tregs), binds a single ligand CCL20. Like the Th1-associated chemokines, CCL20 is inducible and is upregulated in response to the Th17-associated cytokines IL-17A, IL-23 and TNF-α. However, CCL20 is also expressed at high basal levels that initiate an amplification loop in which Th17 cells migrate to tissues in response to CCL20 and then produce IL-17A and IL-23 that further increase CCL20 expression leading to the recruitment of more Th17 cells. Corneal epithelial cells and variety of other mucosal epithelia cell have been found to express CCL20 and Th17-inducing cytokines, such as TGF-β1, IL-6, IL-23 and IL-1β [11]. These factors themselves are able to induce the polarization of a Th17 cell phenotype [18].

In order to investigate the role of chemokine receptors in the migration of CD4^+^ T cells from the regional lymph nodes to the OS, we examined the phenotype of disease in chemokine receptor knock out (KO) mice in our dry eye animal model. We hypothesize that chemokine receptor deficient mice will have less severe disease due to the inability of CD4^+^ T cells to traffic to the ocular surface (OS). Greater understanding of T cell migration to the OS in response to DS may lead to new therapeutic targets (such as chemokine receptor or chemokines) that may halt the amplification loop that results in chronic inflammation and severity of the disease.

## Materials and Methods

### Mice

Six to eight week old female CCR6KO (*B6.129P2-Ccr6^tm1Dgen^/J*), CXCR3KO (*B6.129P2-Cxcr3^tm1Dgen^/J*), RAG1KO (*B6.129S7-Rag1^tm1Mom^/J*), and C57BL/6 were purchased from The Jackson Laboratory (Bar Harbor, ME) for establishment of mouse colonies in our vivarium. All knockout mice have a C57BL/6 background. All animal experiments were approved by the Institutional Animal Care and Use Committee at Baylor College of Medicine and adhered to the Association for Research in Vision and Ophthalmology Statement for the Use of Animals in Ophthalmic and Vision Research.

### Induction of desiccating stress in mice

C57BL/6, CCR6KO, CXCR3KO mice were exposed to desiccating stress (DS). DS was induced by subcutaneous injection of scopolamine hydrobromide (0.5 mg/0.2 ml; Sigma-Aldrich, St. Louis) four times a day (08:00, 12:00, 14:00, and 17:00 h), alternating flanks, for 5 or 10 consecutive days (DS5 or DS10), as previously described [19]–[22]. Mice were placed in a cage with a perforated plastic screen on one side to allow airflow from a fan placed 6 inches in front of it for 16 h/day. Room humidity was maintained at 25–30%. Control mice were maintained in a non-stressed (NS) environment at 50–75% relative humidity without exposure to a forced air draft. DS and NS mice served as donors for adoptive transfer experiments.

### Isolation of murine CD4^+^ T cells

The eyes and lids of mice (*n* = 5 per experiment, in three independent sets of experiments, total of 15 per group, in NS, DS5, and DS10 groups) were excised, pooled, and incubated in 10 ml of 5 mg ml^−1^ Dispase II (Roche Molecular Biochemicals, Indianapolis, IN) in a shaker at 37 °C for 1 h, followed by neutralization with Hank's Buffered Salt Solution (Invitrogen-Gibco, Grand Island, NY) supplemented with 3% fetal bovine serum (Hyclone, Logan, UT). The bulbar and tarsal conjunctivae were scraped with cytology brushes under a dissecting microscope, as previous described [11]. Superficial cervical lymph nodes (CLN) and spleens from donor mice were meshed gently between two frosted end slides, as previously described [23]. Ammonium chloride tris was used to eliminate erythrocytes. Untouched CD4^+^ cells were isolated using magnetic beads according to the manufacturer's instructions (MACS system; Miltenyi Biotec, Auburn, CA). Isolated cells were used in adoptive transfer experiments. Purity of CD4^+^ T cells was determined to be greater than 90% by flow cytometry (data not shown).

### Adoptive transfer experiments

One donor-equivalent of cells was transferred intraperitoneally (i.p.) to T cell deficient recombination activating protein 1 (RAG1KO) mice. One donor-equivalent is defined as the number of cells remaining after the respective *in vitro* manipulation (e.g., CD4^+^ T cells) of a single set of lymph nodes or spleen (approximately 5×10^6^ CD4^+^ cells). The remaining cells represent the total lymphocytes for that splenic or lymph node cell population for a single donor. Experiments were performed three days after adoptive transfer of CD4^+^ T cells.

### Flow Cytometry Analysis

Cells from the epithelial layer of cornea and conjunctiva were pooled after incubation in 5 mg/mL of Dispase II (Roche Molecular Biochemicals, Indianapolis), as previously described [11], and were used to determine the percentage of CD4^+^CCR6^+^ or CD4^+^CXCR3^+^ cells at the ocular surface. Single cells suspensions of the superficial cervical lymph nodes (CLN) were used to determine the percentage of CD4^+^CCR6^+^ or CD4^+^CXCR3^+^ cells in the lymph nodes. FITC- anti-CD4, PE- anti-CXCR3 and Alexa 647- anti-CCR6 antibodies (all from BD Pharmigen) were used. For intracellular staining single-cell suspensions of the spleen and CLN were stimulated with 500 ng/ml phorbol 12-myristate 13-acetate (Sigma) and 500 ng/ml ionomycin (Sigma) plus 1 µl/ml Golgi Stop (BD Pharmigen) for 5 h. Cells were resuspended in fixation-permeabilization solution (Cytofix/Cytoperm) and stained with anti-CD16/32 (to block Fc receptors, BD Pharmigen), followed by cell surface and intracellular staining with FITC- anti-CD4, PE- anti- IL-17A (eBioscience) and APC- anti-IFN-γ antibodies (BD Pharmigen). A narrowing gate strategy was utlized by using a lymphocyte gate, a live/dead cell gate, and finally a CD4^+^ cell gate. Cells found within gate are presented on dot plots. A BD LSRII Benchtop cytometer was used for flow cytometric data acquisition and data was analyzed using BD Diva Software (BD Pharmigen) and Flow Jo software (Tree Star, Inc.), as previously reported [11].

### Corneal Permeability

Corneal epithelial permeability to Oregon green dextran (OGD; 70,000 molecular weight; Invitrogen, Eugene, OR) was assessed by instilling 0.5 µL of OGD onto the ocular surface one minute before euthanasia, as previously described [11]. Corneas were rinsed with PBS and photographed under fluorescence excitation at 470 nm. The severity of corneal OGD staining was graded in digital images in the 2 mm central zone of each cornea by 2 masked observers, using the NIS Elements software (Nikon, Melville, NY).

### Histology and Periodic Acid Schiff Staining and Goblet cell measurement

Enucleated mouse eyes were fixed in 10% formalin, and embedded in paraffin. Six-µm sections were stained with either hematoxylin and eosin or periodic acid-Schiff (PAS) reagent, as previously described [10]. Goblet cell density in the superior and inferior conjunctiva was measured (n = 3/strain/per time point) using NIS Elements Software (version 3.0, BR, Nikon, Melville, NY) using a ×10 objective. Representative images were obtained with a ×40 objective.

### Immunohistochemistry

Immunohistochemistry was performed to detect and count the number of cells in the cornea and conjunctival epithelium that stained positively for CD4 (clone H129.9, 10 µg/mL, BD Bioscience, San Diego, CA) and appropriate biotinylated secondary antibody (BD Pharmingen) and Vectastain Elite ABC using NovaRed reagents (Vector, Burlingame, CA) as previously described [10]. Secondary antibody alone and appropriate anti-rat isotype (BD Biosciences) controls were also examined. Positively stained cells were counted in the goblet cell rich area of the conjunctiva using image-analysis software (NIS Elements Software, Nikon, Melville, NY) using a ×10 objective. Representative images were obtained with a ×40 objective.

### RNA isolation and Real time PCR

Total RNA from conjunctiva, corneal epithelium, and CLN lysates was isolated using a QIAGEN RNeasy Plus Micro RNA isolation kit (Qiagen) following the manufacturer's protocol. After isolation, the concentration of RNA was measured and cDNA was synthesized using the Ready-To-Go™ You-Prime First-Strand kit (GE Healthcare). Real time PCR was performed using specific Taqman probes for Th17 (IL-17A (*IL17A*) (Mm0043918_m1)), Th1 (IFN-γ (*IFNG*) (Mm00801778_m1)), Th-2 (IL-13 (*IL13*) (Mm99999190_m1)) family, IL-6 (*IL6*) (Mm99999064_m1) and MMP-3 (*MMP3*) (Mm00440295_m1) and -9 (*MMP9*) (Mm00442991_m1) genes (Taqman Universal PCR Master Mix AmpErase UNG) in a commercial thermocycling system (StepOnePlus™ Real-Time PCR System, Applied Biosystems), according to the manufacturer's recommendations. The hypoxanthine phosphoribosyltransferase 1(HPRT-1) (*HPRT1*) (Mm00446968_m1) gene was used as an endogenous reference for each reaction. The results of quantitative PCR were analyzed by the comparative C_t_ method in which the target of change = 2^−^ΔΔ^Ct^ and were normalized by the C_t_ value of HPRT-1 and the mean C_t_ of relative mRNA level in the normal control group (non-stressed; NS) of each mouse strain.

### Statistical Analysis

Sample size and power calculations were performed using Statmate software based on preliminary studies. Statistical analyses were performed with Graph Pad Prism software (Graph Pad, Inc, version 5). Data was first evaluated for normality with the Kolmogorov-Smirnov normality test. Appropriate parametric (t-test) or non-parametric (Mann-Whitney U or Wilcoxon) statistical tests were used to make comparisons between 2 groups.

## Results

### Desiccating stress increases the number of CD4^+^ CCR6^+^ and CD4^+^ CXCR3^+^ cells in the regional lymph node and at the ocular surface

**Figure 1:**
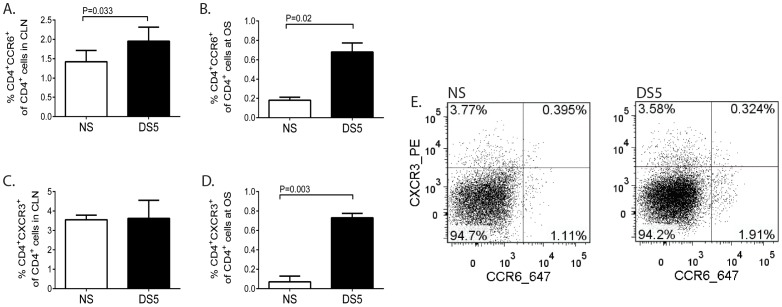
Increased expression of CCR6^+^ and CXCR3^+^ cells in the cervical lymph node and at the ocular surface in response the desiccating stress. **A.** The percentage of CCR6^+^CD4^+^ T cells in the superficial cervical lymph node (CLN) in mice exposed to no stress (NS) or five (DS5) days of desiccating stress (DS) was determined by flow cytometry. The results represent mean±SD of five samples per time point for a total of ten samples (N = 10) in two independent experiments. **B.** The percentage of CCR6^+^CD4^+^ T cells in the ocular surface (OS). The results represent mean±SD of five samples per time point for a total of ten samples (N = 10) in two independent experiments. **C.** The percentage of CXCR3^+^CD4^+^ T cells at the CLN. The results represent mean±SD of five samples per time point for a total of ten samples (N = 10) in two independent experiments. **D.** The percentage of CXCR3^+^CD4^+^ T cells at the OS. The results represent mean±SD of five samples per time point for a total of ten samples (N = 10) in two independent experiments. **E.** A representative dot plot of the percentage of CD4^+^ cells that are CCR6^+^ or CXCR3^+^ in the CLN.

Expression of the chemokine receptors on activated T cells enable them to bind their cognate ligands (chemokines) and mediate their migration to the OS. Th17 and Th1 cells express CCR6 and CXCR3, respectively [24], [25]. Both Th1 and Th17 cells have been reported to be involved in the pathogenesis of dry eye and proliferate in response to desiccating stress [10], [11]. In order to determine if CD4^+^CCR6^+^ and CD4^+^CXCR3^+^ cells are induced in response to DS, total cell populations were isolated from the superficial cervical lymph nodes (CLN) and ocular surface (OS) and analyzed by flow cytometry. In response to DS CD4^+^CCR6^+^cells significantly increased in the CLN (Figure 1A) and OS (Figures 1B and E). Similarly, CD4^+^CXCR3^+^ cells significantly increased at the OS (Figure 1D). We also performed intracellular staining for IL-17A and IFN-gamma and we confirmed that indeed CD4^+^CCR6^+^ produce IL-17A while CD4^+^CXCR3^+^ cells produce, IFN-γ. The percentage of CD4^+^CCR6^+^ cells that are IL-17^+^ is 21.14%±2.28; the percentage of CD4^+^CXCR3^+^ cells that are IFN-γ^+^ is 77.23%±7.93 (data not shown). It is also possible that Th1 cells may express CCR6 or Th17 cells may express CXCR3. However, in this model we could not detect CD4^+^CCR6^+^IFN-γ^+^ cells or CD4^+^CXCR3^+^IL-17A^+^. The percentage of CD4^+^CCR6^+^IFN-γ^+^ cells is 0.058%±0.039. The percentage of CD4^+^CXCR3^+^IL-17A^+^ is 0.048%±0.033 (data not shown). We also determined that both CXCR3KO and CCR6KO mice have similar percentages of CD4^+^IFN-γ^+^ cells and CD4^+^IL-17^+^ cells as wild-type C57BL/6 mice in the CLN (data not shown). In order to address the possibly that CD4^+^ T cells in our model are CD4^+^CXCR3^+^CCR6^+^, the percentage of these double positive cells was determined. We found that the percentage of CD4^+^CXCR3^+^CCR6^+^ cells was 0.347±0.049% for NS animals and 0.362±0.165% for DS5 animals. (Figure 1E).

### Chemokine receptor knock-out mice are resistant to experimental dry eye

**Figure 2:**
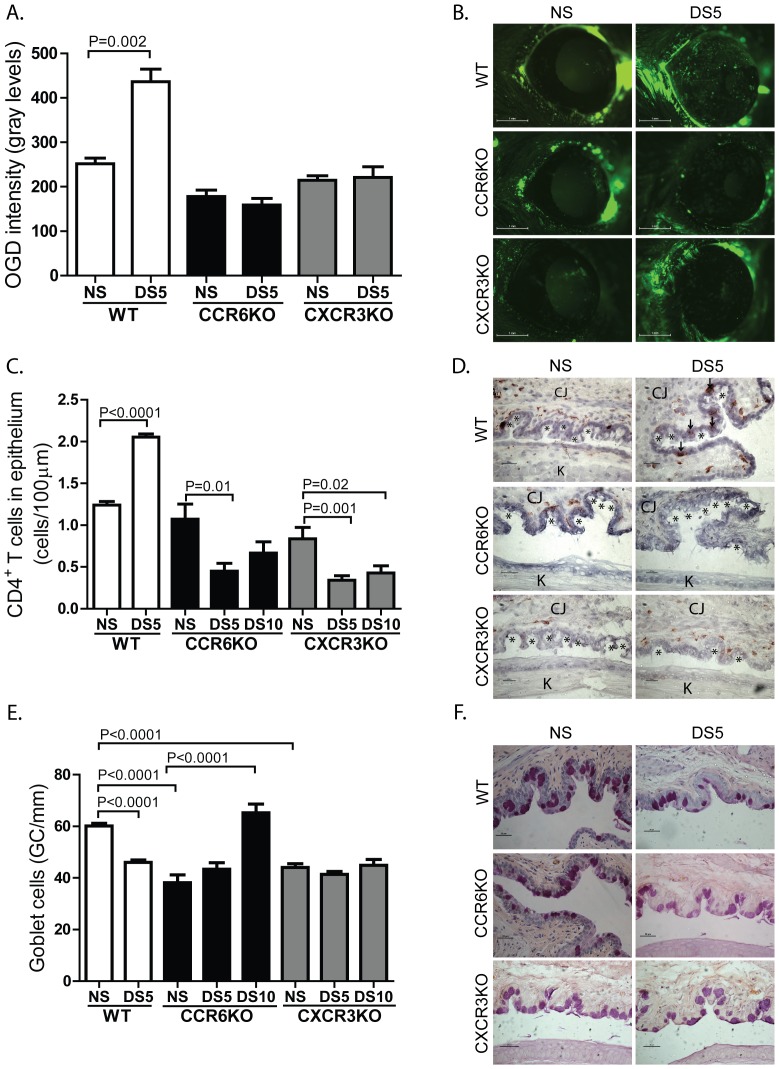
CCR6KO and CXCR3KO mice are resistant to dry eye disease. **A.** Corneal permeability - Mean±SD of Oregon green dextran (OGD) permeability into the corneal epithelium of wild-type (WT), CCR6KO and CXCR3KO mice without (NS) or with 5 days of desiccating stress (DS5). OGD permeability was measured in five to seven mice per strain/time point/experiment. The graph represents the combined results of two independent experiments with a total of 10-14 mice per experimental group. **B.** Representative pictures of OGD staining of each strain. **C.** CD4^+^ T cell density – Mean±SD of the number of CD4^+^ cells per 100 µm of conjunctival epithelium of WT, CCR6KO, and CXCR3KO mice without (NS) or with 5 (DS5) or 10 (DS10) days of desiccating stress. **D.** Representative pictures of CD4^+^ T cell infiltration in each strain. * - indicates goblet cells; ↓ - indicates CD4^+^ T cells; CJ – conjunctiva; K – cornea. **E.** Goblet cell density - Mean±SD of the number of goblet cells per mm of conjunctival epithelium of WT, CCR6KO and CXCR3KO mice without (NS) or with 5 (DS5) or 10 (DS10) days of desiccating stress. Three sections from three animals were examined (N = 9) for each time point/mouse strain for both CD4^+^ T cell and goblet cell density. **F.** Representative pictures of PAS staining of each strain.

The migration of T cells to the OS is dependent on chemokines binding their cognate chemokine receptors on activated T cells. In order to test the hypothesis that ablation of chemokine receptors would ameliorate experimental dry eye, the uptake of OGD as a marker of corneal barrier disruption was measured in non-stressed and CCR6KO and CXCR3KO mice exposed to DS. In response to DS, wild-type C57BL/6 mice had significantly increased uptake of OGD; however, there was no change in OGD staining in CCR6KO or CXCR3KO (Figure 2A and B).

In order to determine if CD4^+^ T cells are able to migrate to the OS in response to DS, the density of CD4^+^ T cells in the conjunctival epithelium was determined by immunohistohemical staining. We have chosen to count infiltrating CD4+ T cells within the conjunctival epithelium at the goblet cell rich area because number of CD4+ T cells in this area inversely correlated with number of goblet cells [10].

As expected, there was a significant increase in the number of CD4^+^ T cells infiltrating the conjunctiva in the goblet cell rich region in WT mice (Figure 2C and D). A significant decrease in density of CD4^+^ T cells was observed in the CCR6KO and CXCR3KO strains after 5 and 10 days of DS (Figure 2C and D). Consistent with disease resistance, histochemical analysis of CCR6KO and CXCR3KO mice revealed a significant increase in the density of conjunctival GC in CCR6KO mice and unchanged density in CXCR3KO in response to DS (Figure 2E and F). Wild-type C57BL/6 mice had a significant decrease in GC density in response to DS (Figure 2E). Thus, the ablation of either CCR6 or CXCR3 renders the mice resistant to experimental dry eye disease.

### IL-17A and IFN-γ expression is absent at the ocular surface of CCR6KO and CXCR3KO mice, however expression is present at the CLN

**Figure 3:**
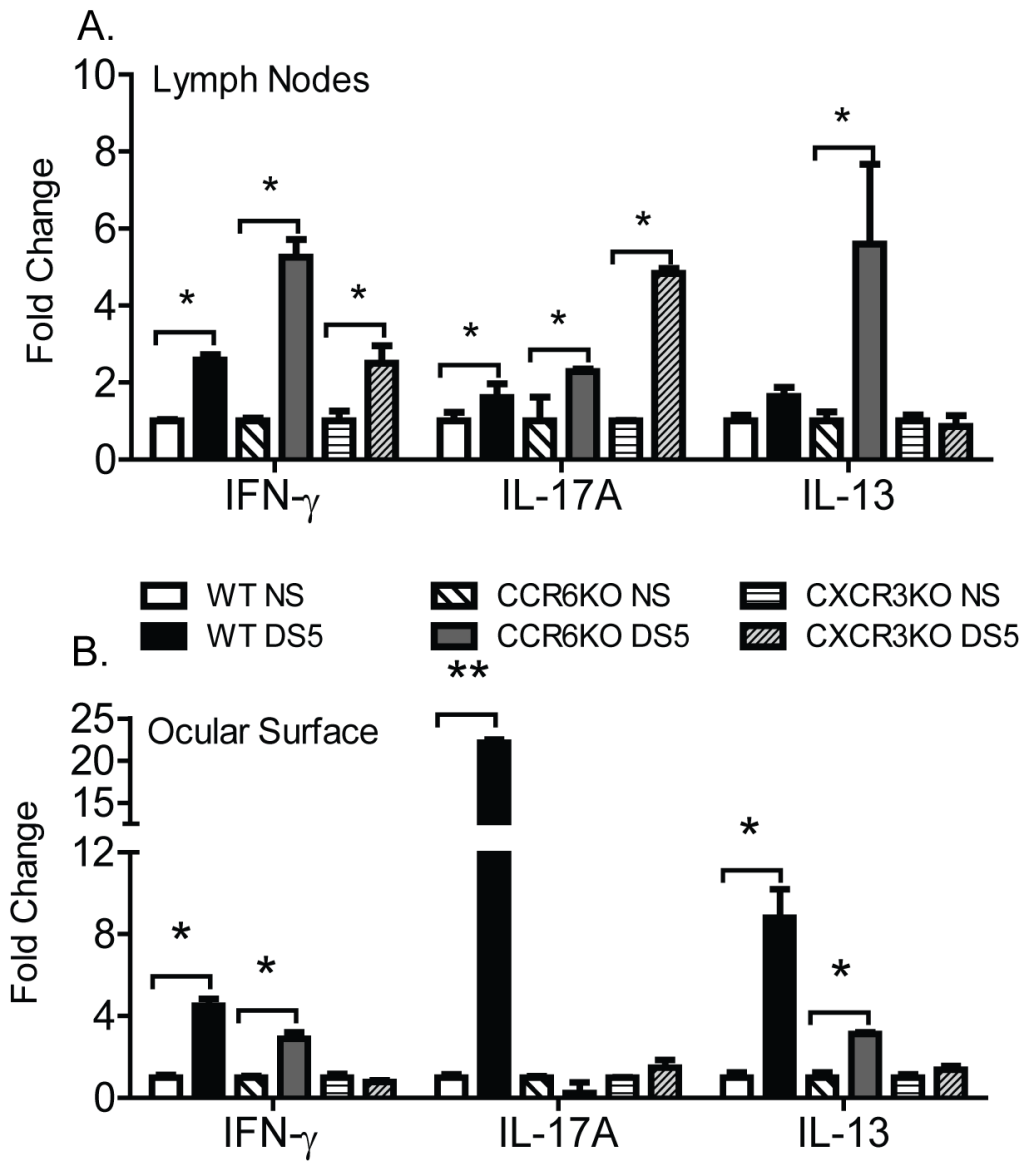
Th1 and Th17 associated cytokines are produced in the cervical lymph node but not the ocular surface. **A.** Gene expression of IFN-γ, IL-17A, and IL-13 in the superficial cervical lymph node (CLN) of wild-type C57BL/6, CCR6KO, and CXCR3KO exposed to DS for 5 days (DS5) or not exposed (NS). **B.** Gene expression of IFN-γ, IL-17A, and IL-13 the ocular surface of wild-type C57BL/6, CCR6KO, and CXCR3KO exposed to DS for 5 days (DS5) or not exposed (NS). Results represent the mean±SD expression 2-3 animals per strain per time point in two independent experiments with a total of five mice. *, P<0.05, **, P<0.01.

We hypothesized that 1) CCR6KO or CXCR3KO CD4^+^ T cells would express cytokines in response to DS in the CLN; 2) in spite of activation and expression of cytokines, CD4^+^ T cells from CCR6KO or CXCR3KO mice would not be able to migrate to the ocular surface. In order to examine the function and activation of CD4^+^ T cells both at CLN and the OS, cells from both sites were collected and gene expression of IFN-γ, IL-17A and IL-13 was examined by quantitative PCR. IL-13 was been shown to play a role in homeostasis of GCs in conjunctiva [26], for this reason IL-13 was also examined. CD4^+^ T cells from CCR6KO mice express mRNA message for IL-17A only in the regional lymph nodes (Figure 3A), but not at the ocular surface in response to DS (Figure 3B). In contrast, IFN-γ and IL-13 was expressed at the OS in CCR6KO exposed to DS, suggesting that Th1 and Th2 cells are still able to migrate to the OS (Figure 3B). Similarly, in response to DS CD4^+^ T cells from CXCR3KO mice expressed IFN-γ at in the CLN (Figure 3A) but not in the eye (Figure 3B). Thus, CD4^+^ T cells from CCR6 and CXCR3KO mice express inflammatory cytokines in response to DS, but cannot migrate to the OS to mediate dry eye disease.

### Adoptively transferred CCR6KO and CXCR3KO CD4^+^ T cells cannot mediate dry eye disease

**Figure 4:**
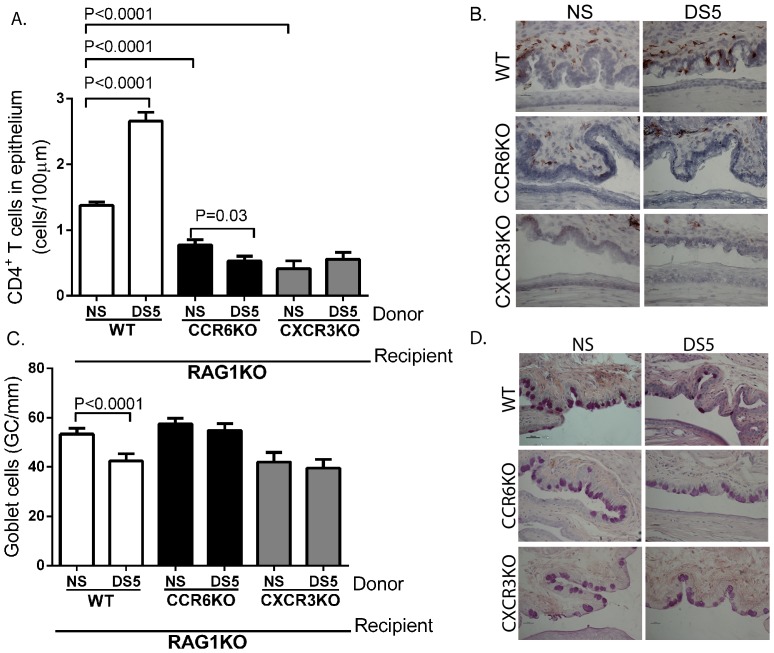
CCR6KO and CXCR3KO CD4^+^ T cells cannot mediate dry eye disease. Wild-type (WT), CCR6KO, or CXCR3KO CD4^+^ T cells from NS or DS5 mice were adoptively transferred to T cell deficient RAG1KO mice. **A.** CD4^+^ T cell density in the conjunctival epithelium of RAG1KO after adoptive transfer. **B.** Representative pictures of CD4^+^ T cell infiltration in each strain after adoptive transfer. **C.** Goblet cell (GC) density in the conjunctival epithelium of RAG1KO after adoptive transfer. Three sections from three animals were examined (N = 9) for each time point/mouse strain for both CD4^+^ T cell and goblet cell density. **D.** Representative pictures of PAS staining of each strain after adoptive transfer.

In order to confirm that the phenotype observed in CCR6KO and CXCR3KO mice was specifically due to ablation of CCR6 and CXCR3 in T cells (not a general immune deficiency), CD4^+^ T cells from CCR6KO or CXCR3KO mice after DS were adoptively transferred to T cell-deficient RAG1KO mice. RAG1KO mice express the chemokines ligands (CCL20, CXCL9, CXCL10, and CXCL11) for CCR6 and CXCR3 at the same levels as wild-type C57Bl/6 mice as determined by qPCR (data not shown). As a control, CD4^+^ T cells from non-stressed animals were also adoptively transferred and compared to DS5 wild-type mice. A significant increase in CD4^+^ T cells and accompanying decrease in conjunctival goblet cells was noted in recipients of CD4+ T cells from DS5 wild-type donors compared to cells from non-stressed mice. There were fewer numbers of CD4^+^ T cells in the conjunctiva between DS5 CCR6KO donors than NS donors (Figure 4A-B). As expected, there is a significant decrease in the number of CD4^+^ T cells migrating to the conjunctival epithelium in mice receiving NS CCR6KO or CXCR3KO CD4^+^ T cells compared to mice that received NS WT CD4^+^ T cells (Figure 4A). Consistent with a decreased disease phenotype, DS5 donor cells from CCR6KO mice did not induce the loss of GCs that was observed in wild-type recipients (Figure 4C-D).

Similarly, CXCR3KO CD4^+^ T cells were also not able to mediate disease. The adoptive transfer of DS5 CXCR3KO CD4^+^ T cells did not induce a significant increase in the density of CD4^+^ T cells in the conjunctiva compared to NS CD4^+^ T cells (Figure 4A-B). As seen with the CCR6KO CD4^+^ T cells, CXCR3KO DS5 CD4^+^ T cells had no effect on GCs density in RAG1KO recipients (Figure 4C-D). Thus, ablation of either CCR6 or CXCR3 prevents CD4^+^ T cells from mediating ocular surface disease.

### Inflammatory cytokines are not expressed in response to DS in the cornea and conjunctiva of CCR6KO and CXCR3KO CD4^+^ T cell recipient mice

**Figure 5:**
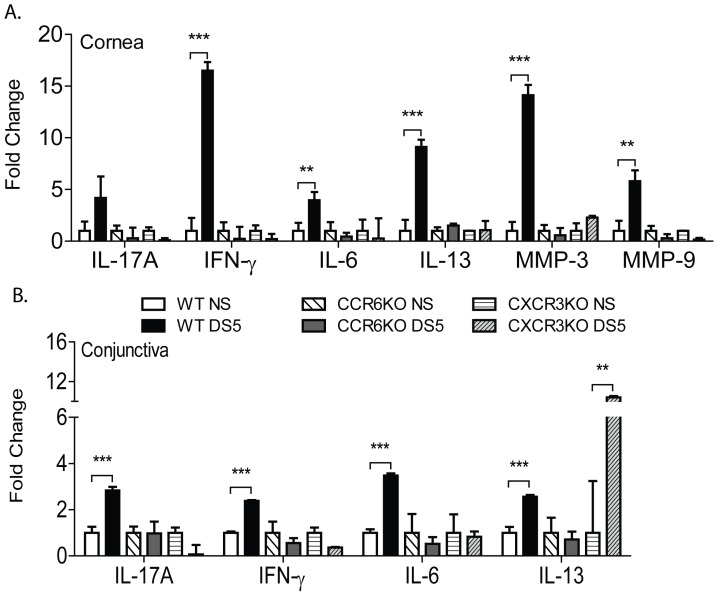
Inflammatory cytokines are not produced in response to DS in the cornea and conjunctiva of CCR6KO and CXCR3KO CD4^+^ T cell recipient mice. Wild-type (WT), CCR6KO, or CXCR3KO CD4^+^ T cells from NS or DS5 mice were adoptively transferred to T cell deficient RAG1KO mice. mRNA analysis for inflammatory cytokines and matrix metalloproteinases (MMP) was performed on the corneal (A) and conjunctival (B) tissues of CD4^+^ T cell RAG1KO recipient mice. Mean±SD of transcript levels of corneal and conjunctival tissues. Results represent the mean±SD expression of 2-3 animals per strain/time point per experiment in two independent experiments for a total of 5-6 mice. *, P<0.05, **, P<0.001, ***, P<0.0001.

In order to further confirm the phenotype of disease in recipient mice that received an adoptive transfer of CCR6 or CXCR3KO CD4^+^ T cells, we initially attempted to perform flow cytometry experiments in the CLN of adoptive transfer recipients. However, the cell recovery was too low to reliably identify the type of Th response. Therefore, we examined expression of inflammatory cytokine and T-cell related cytokines genes at the OS by qPCR. Only recipients of CD4+ T cells from DS5 wild-type donors showed significantly increased expression of IL-17A, IFN-γ, IL-6, MMP-3 and MMP-9 in the cornea (Figure 5A) and IL-17A, IFN-γ, IL-6, and IL-13 in the conjunctiva (Figure 5B). Like mice that received non-stressed wild-type CD4^+^ T cells, mice that received non-stressed or DS5 CD4^+^ T cells from CCR6KO mice had no change in levels of inflammatory cytokines or MMPs in the cornea or conjunctiva (Figure 5A and 5B). Examination of the cornea and conjunctiva of CXCR3KO CD4^+^ T cell recipient mice showed similar results to mice that received CCR6KO CD4^+^ T cells, although a significant increase in IL-13 in the conjunctiva was found in DS5 CXCR3KO CD4^+^ T cell recipients (Figure 5A and 5B). Taken together, these results show that there is a Th-17 and Th-1 response in both cornea and conjunctiva of WT adoptive transfer recipients. While no compensatory Th-1 response was observed in CCR6 recipients, a compensatory Th-2 response is observed in the CXCR3 DS5 recipient mice.

## Discussion

Both CCR6 and CXCR3 play an important role in the recruitment of CD4^+^ T cells in many autoimmune diseases. CCR6^+^ cells have been implicated in the pathogenesis of experimental autoimmune encephalitis, experimental autoimmune uveitis, psoriasis, asthma, and many other diseases [27]–[29]. Similarly, CXCR3^+^ cells are involved in rheumatoid arthritis, systemic lupus erythematosus [13]. Our central hypothesis was that Th1 and Th17 chemokine receptors are needed for the progression of dry eye disease. Our previous studies have demonstrated that an accumulation of Th1 and Th17 cells on the ocular surface occurs in response to desiccating stress [10], [11]. Th1 cells express CXCR3 and Th17 cells express CCR6, and both CD4^+^ CXCR3^+^ cells and CD4^+^ CCR6^+^ cells increase in the regional lymph nodes and at the ocular surface in response to desiccating stress. In spite of cross-regulation *in vitro* [30], [31], both Th1 and Th17 cells concurrently function at the ocular surface to mediate disease. The interaction of CXCR3^+^ Th1 and CCR6^+^ Th17 cells at the ocular surface, whether antagonistic or cooperative, is not understood. This work is relevant in a broader sense in that it provides insight into lymphocyte homing to mucosal tissues and specificity of effector cell responses.

The requirement of chemokine receptors for the migration of T cells from lymphoid tissues to the site of inflammation led us to examine the phenotype of disease in CCR6KO and CXCR3KO mice. Interestingly, both CCR6KO and CXCR3KO mice had less severe disease (decreased OGD intensity, decreased CD4^+^ T cell infiltration, and increased GC density) than wild-type mice. In other disease models, the loss of CCR6 also decreased severity of pathology. Yamazaki et al. demonstrated that lack of CCR6 reduces the severity of experimental autoimmune encephalomyelitis by preventing the migration of Th17 cells [24]. In several disease types, such as Crohn's disease, both Th1 and Th17 cells are important mediators of inflammation [32]. In experimental autoimmune uveitis, Th17 and Th1 cells can each independently induce disease [33]. By contrast, in our model CCR6KO mice presumably still have Th1 cells (as IFN-γ is produced) that could mediate disease on their own, however disease was not observed. Although we found an increase IFN-γ expression (Figure 3B), CCR6KO do not exhibit a decrease in goblet cell density as often found when there is an infiltration of CD4^+^IFN-γ^+^ cells. In fact, we observed an increase in goblet cell density at the DS10 time point (Figure 2 E-F). This suggests that the function of Th1 cells at the ocular surface is dependent on CCR6^+^ Th17 cells. To tease the presence of IFN-γ producing cells, we performed adoptive transfer experiments where only CD4^+^ T cells were transferred into immunodeficient RAG1KO mice. Our results shown in Figure 4 and 5 suggest that there is no compensatory migration of Th1 cells in the CCR6KO CD4^+^ T cell recipient groups.

Similarly, disease was not observed in CXCR3KO mice, which presumably still have Th17 cells. This suggests that both Th1 and Th17 CD4^+^ T cell types are needed to mediate disease at the ocular surface, and their function may be interdependent. For example, Th1 or Th17 may influence the ability of the other at the ocular surface. O’Connor et al. reported that Th1 cells facilitate the entry of Th17 cells into the central nervous system during experimental autoimmune encephalomyelitis [34]. The reverse has also been reported in mice that received a post-vaccination challenge of *Mycobacterium tuberculosis*; Th17 cells were found in the lung prior to Th1 cells and were essential for the accumulation of Th1 cells [35]. Therefore, we hypothesize that the loss of the migratory ability of one type may prevent the function of the other type. For example, the induction of MMPs by CCR6^+^Th17 cells may allow entry of CXCR3^+^Th1 cells in conjunctival or corneal epithelium. It is unclear in our model if Th1 cells are required for recruitment of Th17 cells (or vice versa); however it is clear that both cell types are needed. In agreement, Dohlman et al. recently demonstrated that neutralization of CCL20 in the experimental dry eye reduces the expression of IFN-γ, in addition to reduction of CCR6^+^ Th17 cells, in the conjunctiva [36]. Experiments are currently underway to address this enigma.

CCR6 and CXCR3 can also be expressed on antigen presenting cells (APCs) that present antigen to T cells in lymphoid tissues [13], [37], and they have been reported to be involved in antigen presentation [38], [39]. It has been recently demonstrated that APCs are necessary for the activation of antigen specific lymphocytes in our dry eye model [9]. In order to address the concern that CD4^+^ T cells from CCR6KO or CXCR3KO mice were not functional or not effectively primed, we examined the expression of the prototypical cytokines (IFN-γ, IL-17A and IL-13) of each CD4^+^ T cell subgroup. Our results confirmed that cytokines were expressed in both the lymph nodes and OS in response to DS in wild-type C57BL/6 mice. However, IL-17A was not expressed at the ocular surface in CCR6KO mice (but was produced in the lymph node), and IFN-γ was not expressed at the ocular surface in CXCR3KO mice, but was expressed in CLN. This suggests that cells in CCR6KO and CXCR3KO mice are activated and functional but are not able to migrate to the OS due to the absence of chemokine receptors that induce migration to the ocular surface. Thus, in this model, the ablation of CCR6 or CXCR3 affects the efferent arm of the immune response but not the afferent.

As mentioned above, both CCR6 and CXCR3 are expressed by a number of different cell types. In addition to being expressed by Th17 cells, CCR6 is also expressed by immature dendritic cells, B cells, NKT cells and CD8^+^ T cells [37], [40], [41]. CXCR3 is expressed on natural killer (NK) cells in addition to Th1 cells [25]. Adoptive transfer studies were done in order to determine that the change in disease phenotype was due to CD4^+^ T cells and not another CCR6 or CXCR3 expressing cell type. These studies confirmed that CCR6 or CXCR3 must be expressed on CD4^+^ T cells to mediate disease. Our findings show that a significant increase of CD4^+^ T cells was not observed in recipient mice that received either CCR6KO or CXCR3KO CD4^+^ T cells. Accordingly, the density of goblet cells did not significantly decrease in CCR6KO or CXCR3KO animals. Increased expression of inflammatory cytokines was only observed in mice that received CD4^+^ T cells from wild-type mice, and was not observed in either of the chemokine receptor deficient strains (with the exception of increased IL-13 in recipients of DS5 CXCR3KO). Decreased production of IL-17A, in addition to IFN-γ, was also observed in recipients of CXCR3KO CD4^+^ T cells. Thus, it is possible that the predominant T cell population at the OS in CCR6KO and CXCR3KO mice are Th2 cells, as the T cells present at the OS do not express IL-17A or IFN-γ. Recipients of either CCR6KO or CXCR3KO CD4^+^ T cells also failed to upregulate MMP-3 or -9 in the cornea in response to DS, which may limit the ability of Th1 or Th17 cells to infiltrate the cornea. Overall, these results suggest that there is no compensatory migration of Th1, Th17 or Th2 cells in the CCR6KO or CXCR3KO recipient groups. The interaction of Th1 and Th17 cells are the ocular surface is poorly understood and warrants further investigation.

The requirement of chemokine receptors for the expression of dry eye disease may present a viable therapeutic target for treatment. As mentioned above, the chemokine ligands for CCR6 (CCL20) and CXCR3 (CXCL9, -10, -11) are upregulated in response to desiccating stress [11], [15], [17]. It has been suggested that CCR6/CCL20 could be a potential therapeutic target for psoriasis [42]. The accessibility of the OS for treatment with inhibitors or antibodies to block the CCR6/CCL20 or CXCR3/CXCL9, -10, -11 interactions may prove to be very effective in limiting the amplification loop that results in the inflammation associated with dry eye disease.
